# Investigation of optimum ohmic heating conditions for inactivation of *Escherichia coli* O157:H7, *Salmonella enterica* serovar Typhimurium, and *Listeria monocytogenes* in apple juice

**DOI:** 10.1186/s12866-017-1029-z

**Published:** 2017-05-19

**Authors:** Il-Kyu Park, Jae-Won Ha, Dong-Hyun Kang

**Affiliations:** 1 Department of Food and Animal Biotechnology, College of Agricultural Biotechnology, Center for Food and Bioconvergence, and Institute of GreenBio Science & Technology, Research Institute for Agricultural and Life Sciences, Seoul National University, Seoul, 08826 Korea; 2 Department of Food and Biotechnology, College of Engineering, Food & Bio-industry Research Center, Hankyong National University, Anseong-si, 17579 Korea

**Keywords:** Ohmic heating, Apple juice, System performance efficiency, Foodborne pathogen, Inactivation

## Abstract

**Background:**

Control of foodborne pathogens is an important issue for the fruit juice industry and ohmic heating treatment has been considered as one of the promising antimicrobial interventions. However, to date, evaluation of the relationship between inactivation of foodborne pathogens and system performance efficiency based on differing soluble solids content of apple juice during ohmic heating treatment has not been well studied. This study aims to investigate effective voltage gradients of an ohmic heating system and corresponding sugar concentrations (°Brix) of apple juice for inactivating major foodborne pathogens (*E. coli* O157:H7, *S*. Typhimurium, and *L. monocytogenes*) while maintaining higher system performance efficiency.

**Results:**

Voltage gradients of 30, 40, 50, and 60 V/cm were applied to 72, 48, 36, 24, and 18 °Brix apple juices. At all voltage levels, the lowest heating rate was observed in 72 °Brix apple juice and a similar pattern of temperature increase was shown in18–48 °Brix juice samples. System performance coefficients (SPC) under two treatment conditions (30 V/cm in 36 °Brix or 60 V/cm in 48 °Brix juice) were relatively greater than for other combinations. Meanwhile, 5-log reductions of the three foodborne pathogens were achieved after treatment for 60 s in 36 °Brix at 30 V/cm, but this same reduction was observed in 48 °Brix juice at 60 V/cm within 20 s without affecting product quality.

**Conclusions:**

With respect to both bactericidal efficiency and SPC values, 60 V/cm in 48 °Brix was the most effective ohmic heating treatment combination for decontaminating apple juice concentrates.

## Background

The U.S. Food and Drug Administration stated that the possibility for contamination with foodborne pathogens is low in foods with pH below 4.6 [1]. However, acidic foods such as fruit juice have emerged as a novel substrate in which foodborne pathogens can maintain their viability since several illness outbreaks involving them have been documented [2]. Major foodborne pathogens implicated in fruit juice-borne outbreaks are *Escherichia coli* O157:H7 and *Salmonella enterica* serovar Typhimurium [3]. In the United States in 1996, a serious foodborne outbreak occurred in which one person died and 70 people were infected with *E. coli* O157:H7 traced to apple cider [4]. A multistate outbreak caused by *S*. Typhimurium was reported in the United States in 2005 which was associated with consumption of orange juice [5]. *Listeria monocytogenes* is a Gram positive bacterium and has acid tolerance as do *E. coli* O157:H7 and *S*. Typhimurium [6]. Although outbreaks of foodborne illnesses linked to *L. monocytogenes* have not occurred in fruit juices, the National Advisory Committee on Microbiological Criteria for Foods suggested that *L. monocytogenes* should be categorized as a target bacterium even though no association has been identified between *L. monocytogenes* and fruit juices [7]. Apples used for producing juice can become contaminated with these pathogens from several sources, such as apples in orchards that have fallen onto the ground, contamination with manure, or those insufficiently washed [8, 9].

The U.S. Food and Drug Administration has regulated that facilities for pasteurization should ensure a minimum of 5-log pathogen reduction [10]. Thermal methods such as hot water or steam traditionally have been used to pasteurize apple juice. Although conventional heating guarantees food microbiological safety, it causes deterioration of overall quality involving nutritional degradation, color change, and flavor loss [11, 12]. Novel technologies such as radio frequency, microwave, and ohmic heating have emerged as alternatives in order to compensate for the drawbacks of traditional heating. Ohmic heating among innovative thermal technologies is an appropriate system to use for fruit juice pasteurization in that it is able to heat rapidly and uniformly with high temperature for a short time (HTST process) and is amenable to a continuous type design [13, 14]. Ohmic heating is a technology where heat is internally generated by the passage of alternating electric current in which foods act as a resistor [15], and the heating rate in ohmic heating is related to the electrical conductivity of liquid food products [14]. Because of this characteristic, many food engineers have studied ohmic heating associated with the electrical properties of foods. Castro et al. [16] studied the relationship between temperature and sugar content on the electrical conductivity of strawberry products during ohmic heating. Also, Icier and Ilicali [17] investigated the effect of orange juice concentration on system performance efficiency during ohmic heating. Therefore, not only the degree of antimicrobial effect but also several other factors such as the concentration of dissolved solids concerned with system performance efficiency should be considered in order to apply an ohmic heating pasteurization system practically by the fruit juice industry. To date, evaluation of the relationship between inactivation of foodborne pathogens and system performance efficiency based on differing soluble solids content of juices during ohmic heating treatment has not been well studied.

The purpose of this research was to investigate the optimum sugar concentration (°Brix) of apple juice and corresponding voltage gradient of an ohmic heating system for achieving both effective inactivation of foodborne pathogens including *E. coli* O157:H7, *S*. Typhimurium, and *L. monocytogenes* and higher system performance efficiency.

## Methods

### Bacterial strains and culture preparation

All bacterial strains, namely, *E. coli* O157:H7 (ATCC 35150, ATCC 43889, and ATCC 43890), *S*. Typhimurium (ATCC 19585, ATCC 43971, and DT 104) and *L. monocytogenes* (ATCC 19114, ATCC 19115, ATCC 15313) were obtained from the Bacterial Culture Collection at Seoul National University (Seoul, South Korea) and used for all experiments. All strains were stored at −80 °C in 0.7 ml of Tryptic Soy Broth (TSB; Difco Becton Dickinson, Sparks, MD, USA) and 0.3 ml of 50% glycerol (vol/vol). Working cultures were streaked onto Tryptic Soy Agar (TSA; Difco), incubated at 37 °C for 24 h, and stored at 4 °C. Each strain of *E. coli* O157:H7, *S*. Typhimurium, and *L. monocytogenes* was cultured in 5 ml TSB for 24 h at 37 °C, harvested by centrifugation at 4000 × *g* for 20 min at 4 °C, and washed three times with 0.2% peptone water (PW, Difco). The final pellets were resuspended in 0.2% PW, corresponding to approximately 10^8^ ~ 10^9^ CFU/ml. Subsequently, suspended pellets of each strain of the three pathogens were mixed to produce a culture cocktail.

### Sample preparation and inoculation

Pasteurized apple juice concentrate (pH 3.5, 72 °Brix), free of any preservatives, was purchased from a local grocery store (Incheon, Korea). Apple juice concentrate was diluted with sterile distilled water to 48, 36, 24, and 18 °Brix. Sugar concentration (°Brix) was measured by a digital refractometer (Atago co.,Ltd., Japan). Then, a 0.2-ml aliquot of the mixed culture cocktail (*E. coli* O157:H7, *S*. Typhimurium, and *L. monocytogenes*) was inoculated into each 25 ml sample of apple juice of different solids content. The final cell concentration was ca. 10^6^ ~ 10^7^ CFU/ml.

### Experimental apparatus

**Fig. 1:**
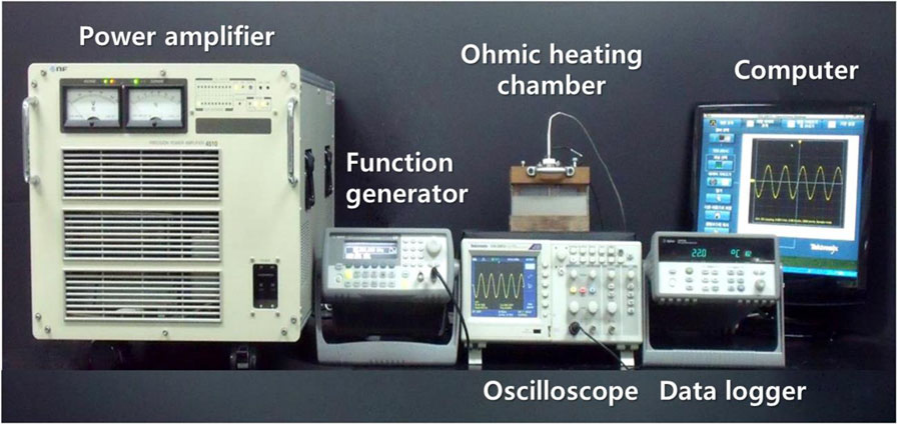
Ohmic heating system at Seoul National University (Seoul, Korea)

Ohmic heating treatments were conducted in a previously described apparatus [18]. The experimental device (Fig. 1) consisted of a two-channel digital storage oscilloscope (TDS2001C; Tektronix, Inc., Beaverton, CO), a precision power amplifier (4510; NF corp., Yokohama, Japan), a function generator (33210A; Agilent Technologies, Palo Alto, CA), a data acquisition instrument (34,790 A; Agilent Technologies), and an ohmic heating chamber. In the middle of a rectangular container (an ohmic heating chamber, 2 by ×15 by ×6 cm) consisting of component Pyrex glass, two titanium electrodes and a K-type thermocouple coated with Teflon were located. The distance between the cross-sectional area and the two titanium electrodes was 2 cm and 60 cm^2^, respectively. Multiple waveforms such as sine, square, ramp, pulse, triangle, noise, and custom waveforms could be produced by the function generator which permitted a frequency range of 1 MHz to 10 MHz and a maximum output signal of 5 V. These signals were expanded by the power amplifier from 45 to 20 kHz and a maximum output of 141 VAC. Each titanium electrode received signals amplified by the power amplifier. The signals, including waveform, frequency, voltage, and current, were measured using the two-channel digital storage oscilloscope. The data acquisition instrument was used to obtain temperature histories in this study.

### Ohmic heating treatment

The ohmic heating chamber was filled with 25 ml of sample for treatment. A 20 kHz frequency and sine waveform were utilized in all experiments. Since electrochemical reactions can occur at standard line voltage frequency (60 Hz) during ohmic heating and it may affect inactivation of foodborne pathogens [18, 19], 20 kHz, a high frequency that does not cause electrochemical reactions, was chosen in this study. For obtaining temperature and electric current data, treatments were conducted at a fixed 30, 40, 50, and 60 V/cm setting in apple juice of 72, 48, 36, 24, and 18 °Brix for 90 s. Temperature and electric current were recorded every 1 s. For microbial inactivation experiments, inoculated samples were treated at a fixed 30 or 60 V/cm setting in 72, 48, 36, 24, and 18 °Brix apple juice for 0, 10, 20, 30, 40, 50, and 60 s.

### Bacterial enumeration

For enumeration of bacteria, each treated 25 ml sample was immediately transferred into a sterile stomacher bag (Labplas Inc., Sainte-Julie, Quebec, Canada) containing 225 ml of iced 0.2% PW (maintained on crushed ice) and homogenized for 2 min with a stomacher (Easy Mix, AES Chemunex, Rennes, France). One ml aliquots of homogenized samples were tenfold serially diluted in 9 ml of 0.2% PW, and 0.1 ml of sample or diluent was spread-plated onto each selective medium. For the enumeration of *E. coli* O157:H7, *S*. Typhimurium and, *L. monocytogenes*, Sorbitol MacConkey agar (SMAC; Difco), Xylose Lysine Desoxycholate agar (XLD; Difco) and Oxford Agar Base (OAB; Difco) with antimicrobic supplement (Bacto™ Oxford Antimicrobic Supplement, Difco) were used as selective media, respectively. Where low numbers of surviving cells were anticipated, 250 μl of sample was spread-plated onto each of four plates to lower the detection limit (detection limit = 10 CFU/g). All agar media were incubated at 37 °C for 24–48 h before counting. To confirm the identity of the pathogens, colonies were selected randomly from the enumeration plates and subjected to serological or biochemical tests [*E. coli* O157:H7 latex agglutination assay (RIM, Remel, Lenexa, KS, USA), *Salmonella* latex agglutination assay (Oxoid, Ogdensberg, NY, USA), and API *Listeria* (bioMérieux, Inc. Hazelwood, MO, USA)].

### System performance coefficient measurement

The system performance coefficient (SPC) of ohmic heating was determined from temperature, voltage, and current data [17] and calculated as follows (equation 1):

1 $$ SPC=\frac{mCp\Delta T}{\sum \Delta VIt} $$

Where *m* is mass (g), *Cp* is specific heat capacity (J/g K), ∆*T* is difference between final temperature and initial temperature (K), ∆*V* is voltage applied (V), *I* is electric current (A), and *t* is time (s). ∑Δ*VIt* is the energy given to the system, *mCp*Δ*T* is energy given to the system minus energy loss during ohmic heating. The ratio of *mCp*Δ*T* to ∑Δ*VIt* indicates the system performance coefficient [17].

### Color and pH measurement

To assess color changes of treated apple juice, a Minolta colorimeter (CR400; Minolta Co., Osaka, Japan) was used in this study. Color of apple juice were expressed by values of L*, a*, and b* (color lightness, redness, and yellowness, respectively) [20]. A pH meter (Seven Multi 8603; Mettler Toledo, Greifensee, Switzerland) was utilized to measure pH values.

### Statistical analysis

All experiments were conducted three times with duplicate samples. Data were analyzed by the ANOVA procedure of SAS (Version 9.2. SAS Institute Inc., NC, USA), and mean values were separated using Tukey-Kramer’s multiple range test. A *P* value of <0.05 was used to indicate significant differences.

## Results and discussion

### Temperature profiles of different concentrations of apple juice

**Fig. 2:**
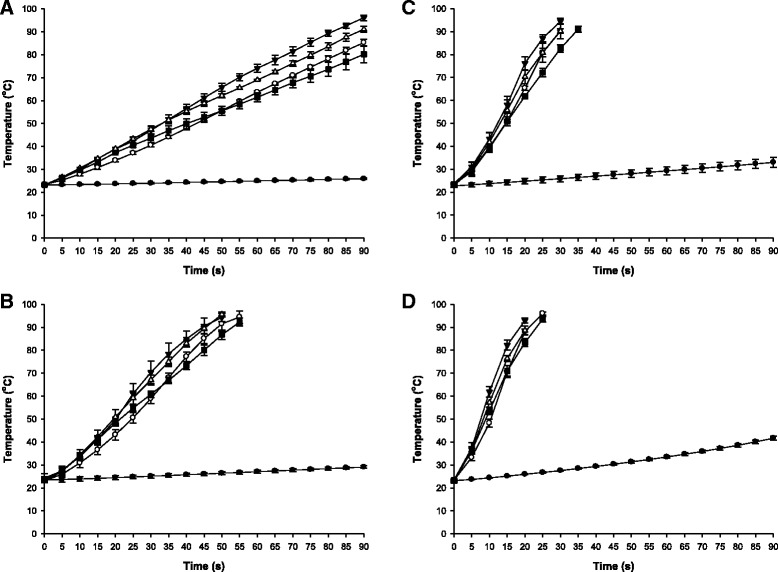
Temperature-time curves for apple juice of 18 (■), 24 (△), 36 (▼), 48 (○), and 72 (●) °Brix during ohmic heating at voltage gradients of 30 V/cm (**a**), 40 V/cm (**b**), 50 V/cm (**c**), and 60 V/cm (**d**). Error bars indicate standard deviations calculated from triplicates

There are various factors affecting electrical conductivity of liquids. Electrical conductivity relies on chemical components, ion activity, and viscosity of liquids. Such an electrical characteristic, along with juice concentration, could have an influence on temperature rise and microbial inactivation [17, 21]. A study by Palaniappan and Sastry [22] stated that the relationship between electrical conductivity and temperature was linear but conductivity decreased with increasing soluble solids content in tomato and orange juices. The results of the present study were also consistent with previous reports. The heating rates of various concentrations of apple juice during ohmic heating at different voltage gradients are shown in Fig. 2. Temperature rise was more rapid in higher concentrations than in lower concentrations of juice up to 36 °Brix. However, when approaching 48 °Brix, the rate of temperature increase began to decline. The slowest rate of temperature increase was observed at the maximum sugar concentration (72 °Brix) of apple juice since electric conductivity was suppressed as sugar concentration approached the maximum levels included in this study (data not shown).

### System performance efficiency at different concentrations of apple juice and voltage gradients

**Fig. 3:**
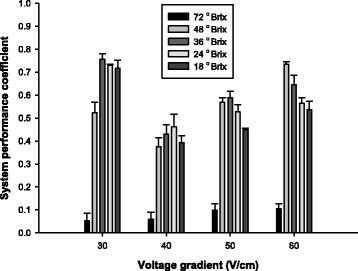
System performance coefficient levels for 18, 24, 36, 48, and 72 °Brix apple juice during ohmic heating at voltage gradients of 30, 40, 50, and 60 V/cm. Error bars indicate standard deviations calculated from triplicates

The system performance coefficient (SPC), which affects processing cost, was considered as an important factor in this study. Icier and Ilicali [17] reported that SPC values of ohmic heating depended strongly on the voltage gradient applied to orange juice concentrates. For the 60 V/cm voltage gradients SPCs were approximately 0.52–0.59, which indicated that 41–48% of the electrical energy applied to the system was not used in heating orange juice concentrates. For low voltage gradients (20 V/cm), the conversion of electrical energy into heat was greater. A similar tendency was also observed in the present study. Figure 3 shows system performance coefficients of ohmic heating at different sample concentrations and voltage gradients. Average SPC values at 40, 50, and 60 V/cm were not as high as that of 30 V/cm. The energy loss at a voltage gradient of 30 V/cm was the lowest when 36 °Brix apple juice was subjected to ohmic heating, which indicated that ca. 75% of the electrical energy applied to the system was utilized for heating (Fig. 3). When treated with 40 V/cm, the worst system performance efficiencies were detected at all sample concentrations. As applied voltage increased, overall SPC gradually increased from 40 to 60 V/cm. Following higher voltage gradients (60 V/cm), the peak system efficiency was observed in 48 °Brix juice. The SPC value for 48 °Brix apple juice at 60 V/cm, which is the actual electrical energy used to heat the samples, was ca. 73%. In the case of 72 °Brix apple juice, SPC values were absolutely lower than in any other concentration of apple juice (Fig. 3). This can be correlated to electrical conductivity or resistance of juice at higher sugar concentrations.

### Effect of ohmic heating for inactivation of foodborne pathogens at different voltage gradients

Control of foodborne pathogens is an important issue for the fruit juice industry and ohmic heating treatment has been considered as one of the promising antimicrobial interventions. In our previous study [18], reduction of *E. coli* O157:H7, *S.* Typhimurium, and *L. monocytogenes* resulting from ohmic heating was significantly higher (*P* < 0.05) than that resulting from conventional heating at equal temperatures of 55, 58, and 60 °C in apple juice. These results showed that electric field-induced ohmic heating led to additional bacterial inactivation due not only to thermal effect but also to electroporation-caused cell damage [18]. As the latest in a series of research studies on ohmic heating of apple juice, we attempted to optimize the processing conditions of ohmic heating based on system performance efficiency and inactivation level of pathogens to provide a practical methodology for the fruit juice industry.

Tables 1, 2 and 3 shows the reduction of *E. coli* O157:H7, *S*. Typhimurium, and *L. monocytogenes* in different apple juice concentrations during ohmic heating, respectively. At 30 V/cm, ohmic heating for 60 s achieved 0.95, 2.59, 6.78, 5.21, and 2.71 log reductions of *E. coli* O157:H7 in 72, 48, 36, 24, and 18 °Brix apple juice, respectively. Also, reductions of 1.40, 2.88, 6.71, 6.70, and 3.27 log CFU/ml in concentrations of 72, 48, 36, 24, and 18 °Brix, respectively, were observed in *S*. Typhimurium. In the case of *L. monocytogenes*, levels of log reduction following ohmic heating were 0.47, 1.74, 5.01, 3.91, and 1.13, respectively, in juice concentrations of 72, 48, 36, 24, and 18 °Brix. From these results at 30 V/cm, maximum log reductions of the three foodborne pathogens were observed in 36 °Brix apple juice. Dramatic levels of inactivation were achieved in 18–48 °Brix apple juice during ohmic heating at 60 V/cm. Reductions of *E. coli* O157:H7 were 6.32, 6.58, 6.88, and 6.93 log CFU/ml in 48, 36, 24, and 18 °Brix juice, respectively, after ohmic heating for 20 s. Similarly, ohmic heating for 20 s accomplished 5.80, 6.10, 6.60, and 6.68 log reductions of *S*. Typhimurium in 48, 36, 24, and 18 °Brix juice, respectively. Log reductions of 5.71, 5.70, 5.82, and 5.93 in 48, 36, 24, and 18 °Brix apple juice, respectively, were observed for *L. monocytogenes*. Thus, the time duration required for 5-log reduction at 30 V/cm in 36 °Brix apple juice was three times longer than for 60 V/cm at all apple juice concentrations with the exception of 72 °Brix. Also, commercial processing of higher concentration apple juice has the advantage of greater production yield (of 18 °Brix juice). Therefore, with respect to bactericidal efficiency, SPC values, and treatment time, ohmic heating application of 60 V/cm in 48 °Brix apple juice could be more efficient than that of 30 V/cm in 36°Brix.

**Table 1:** Log reductions of *E. coli* O157:H7 in 72, 48, 36, 24, and 18 °Brix apple juice subjected to ohmic heating at 30 and 60 V/cm

Voltage		Log reduction [log_10_ (N_0_/N)]^a^ by treatment time (s)													
gradient	°Brix	0		10		20		30		40		50		60	
30 V/cm	72	0.00 ± 0.00	A	0.44 ± 0.22	B	0.42 ± 0.05	B	0.53 ± 0.16	B	0.56 ± 0.32	B	0.71 ± 0.22	BC	0.95 ± 0.03	C
	48	0.00 ± 0.00	A	0.26 ± 0.07	A	0.34 ± 0.02	A	0.41 ± 0.05	A	0.89 ± 0.22	B	1.38 ± 0.22	C	2.59 ± 0.63	D
	36	0.00 ± 0.00	A	0.28 ± 0.14	A	0.34 ± 0.04	A	0.91 ± 0.37	B	1.40 ± 0.30	C	3.33 ± 0.31	D	6.78 ± 0.11	E
	24	0.00 ± 0.00	A	0.24 ± 0.08	AB	0.22 ± 0.13	AB	0.71 ± 0.48	BC	1.26 ± 0.46	C	2.97 ± 0.47	D	5.21 ± 0.36	E
	18	0.00 ± 0.00	A	0.09 ± 0.08	A	0.17 ± 0.30	AB	0.17 ± 0.06	AB	0.67 ± 0.31	B	1.34 ± 0.50	C	2.71 ± 0.29	D
60 V/cm	72	0.00 ± 0.00	A	0.38 ± 0.22	AB	0.67 ± 0.22	B	0.62 ± 0.06	B	0.55 ± 0.29	AB	0.73 ± 0.32	B	0.73 ± 0.60	B
	48	0.00 ± 0.00	A	0.42 ± 0.13	B	6.33 ± 0.13	C	ND		ND		ND		ND	
	36	0.00 ± 0.00	A	0.82 ± 0.17	B	6.58 ± 0.22	C	ND		ND		ND		ND	
	24	0.00 ± 0.00	A	0.74 ± 0.58	B	6.88 ± 0.06	C	ND		ND		ND		ND	
	18	0.00 ± 0.00	A	0.50 ± 0.41	B	6.93 ± 0.11	C	ND		ND		ND		ND	

^a^The values are means ± standard deviations from three replications. Values in the same row followed by the same letter are not significantly different (*P* > 0.05). *ND* not detected.

**Table 2:** Log reductions of *S.* Typhimurium in 72, 48, 36, 24, and 18 °Brix apple juice subjected to ohmic heating at 30 and 60 V/cm

Voltage		Log reduction [log_10_ (N_0_/N)]^a^ by treatment time (s)													
gradient	°Brix	0		10		20		30		40		50		60	
30 V/cm	72	0.00 ± 0.00	A	0.64 ± 0.30	AB	0.72 ± 0.28	B	1.23 ± 0.28	B	0.90 ± 0.20	B	1.06 ± 0.77	B	1.40 ± 0.44	B
	48	0.00 ± 0.00	A	0.52 ± 0.21	B	0.33 ± 0.15	AB	0.69 ± 0.18	BC	1.08 ± 0.30	C	1.86 ± 0.54	D	2.88 ± 0.48	E
	36	0.00 ± 0.00	A	0.29 ± 0.06	AB	0.43 ± 0.15	B	0.80 ± 0.08	C	1.62 ± 0.25	D	3.99 ± 0.33	E	6.71 ± 0.13	F
	24	0.00 ± 0.00	A	0.22 ± 0.19	A	0.31 ± 0.14	A	0.93 ± 0.26	B	1.81 ± 0.25	C	3.42 ± 0.50	D	6.70 ± 0.16	E
	18	0.00 ± 0.00	A	0.09 ± 0.10	A	0.17 ± 0.11	AB	0.48 ± 0.23	B	0.87 ± 0.07	C	1.44 ± 0.22	D	3.27 ± 0.40	E
60 V/cm	72	0.00 ± 0.00	A	0.61 ± 0.12	AB	0.90 ± 0.30	B	1.00 ± 0.51	B	1.06 ± 0.54	B	1.26 ± 0.30	B	1.20 ± 0.66	B
	48	0.00 ± 0.00	A	0.42 ± 0.44	A	5.81 ± 0.06	B	ND		ND		ND		ND	
	36	0.00 ± 0.00	A	0.83 ± 0.32	B	6.10 ± 0.24	C	ND		ND		ND		ND	
	24	0.00 ± 0.00	A	0.79 ± 0.88	A	6.61 ± 0.13	B	ND		ND		ND		ND	
	18	0.00 ± 0.00	A	0.65 ± 0.45	B	6.68 ± 0.14	C	ND		ND		ND		ND	

^a^The values are means ± standard deviations from three replications. Values in the same row followed by the same letter are not significantly different (*P* > 0.05). *ND* not detected.

**Table 3:** Log reductions of *L. monocytogenes* in 72, 48, 36, 24, and 18 °Brix apple juice subjected to ohmic heating at 30 and 60 V/cm

Voltage		Log reduction [log_10_ (N_0_/N)]^a^ by treatment time (s)													
gradient	°Brix	0		10		20		30		40		50		60	
30 V/cm	72	0.00 ± 0.00	A	0.34 ± 0.15	A	0.32 ± 0.16	A	0.34 ± 0.06	A	0.32 ± 0.22	A	0.37 ± 0.32	A	0.47 ± 0.49	A
	48	0.00 ± 0.00	A	0.21 ± 0.08	AB	0.36 ± 0.13	B	0.52 ± 0.14	BC	0.73 ± 0.30	C	1.34 ± 0.12	D	1.74 ± 0.24	E
	36	0.00 ± 0.00	A	0.43 ± 0.20	B	0.42 ± 0.14	B	0.67 ± 0.19	B	1.10 ± 0.18	C	1.90 ± 0.22	D	5.01 ± 0.35	E
	24	0.00 ± 0.00	A	0.04 ± 0.08	A	0.13 ± 0.12	A	0.21 ± 0.13	A	0.74 ± 0.20	B	1.18 ± 0.37	C	3.91 ± 0.26	D
	18	0.00 ± 0.00	A	0.07 ± 0.13	A	0.04 ± 0.10	A	0.27 ± 0.19	A	0.23 ± 0.28	A	0.42 ± 0.24	A	1.13 ± 0.41	B
60 V/cm	72	0.00 ± 0.00	A	0.31 ± 0.14	A	0.23 ± 0.12	A	0.33 ± 0.19	A	0.40 ± 0.31	A	0.40 ± 0.25	A	0.38 ± 0.40	A
	48	0.00 ± 0.00	A	0.57 ± 0.28	B	5.71 ± 0.27	C	ND		ND		ND		ND	
	36	0.00 ± 0.00	A	1.46 ± 0.09	B	5.71 ± 0.23	C	ND		ND		ND		ND	
	24	0.00 ± 0.00	A	0.50 ± 0.42	A	5.83 ± 0.13	B	ND		ND		ND		ND	
	18	0.00 ± 0.00	A	0.66 ± 0.11	B	5.94 ± 0.20	C	ND		ND		ND		ND	

^a^The values are means ± standard deviations from three replications. Values in the same row followed by the same letter are not significantly different (*P* > 0.05). *ND* not detected.

### The influence of ohmic heating on quality of apple juice

Additionally, ohmic heating is a suitable technology for minimizing degradation of juice quality due to the fundamental property of ohmic heating, which generates internal heat in food materials [14]. Color and pH values of 18, 24, 36, 48, and 72 °Brix apple juice following ohmic heating at 30 and 60 V/cm are shown in Table 4. All experiments were limited to a maximum treatment time of 60 s. In case of 60 V/cm, treatment time was restricted to 20 s in 18, 24, 36, and 48 °Brix apple juice because 20 s was a sufficient time interval for obtaining the target microbial reductions. L*, a*, and b* values of samples treated versus not treated with ohmic heating were not significantly (*P* > 0.05) different. The pH values of treated samples did not significantly differ from those of non-treated samples. Thus, the proposed parameters for optimal ohmic heating did not significantly affect the quality of apple juice product (Table 4).

**Table 4:** Color values^b^ and pH of treated and untreated apple juice of 18, 24, 36, 48, and 72 °Brix at 30 and 60 V/cm following ohmic heating

Voltage gradient	Mean ± SD^a^					
	Solids content (°Brix)	Treatment time (s)	pH	Color^b^		
				L*	a*	b*
30 V/cm	72	0	3.42 ± 0.00	26.47 ± 0.06	0.38 ± 0.01	4.11 ± 0.01
		60	3.42 ± 0.01	26.44 ± 0.08	0.38 ± 0.02	4.09 ± 0.05
	48	0	3.51 ± 0.00	25.43 ± 0.29	0.49 ± 0.03	4.66 ± 0.09
		60	3.51 ± 0.01	25.47 ± 0.72	0.47 ± 0.11	4.56 ± 0.22
	36	0	3.54 ± 0.00	24.85 ± 0.10	0.49 ± 0.04	5.16 ± 0.05
		60	3.54 ± 0.01	24.76 ± 0.19	0.54 ± 0.02	5.05 ± 0.19
	24	0	3.57 ± 0.01	24.72 ± 0.65	0.32 ± 0.04	5.02 ± 0.35
		60	3.57 ± 0.01	24.55 ± 0.08	0.38 ± 0.01	5.46 ± 0.09
	18	0	3.59 ± 0.00	24.23 ± 0.23	0.25 ± 0.02	5.30 ± 0.43
		60	3.60 ± 0.00	24.51 ± 0.19	0.27 ± 0.02	5.58 ± 0.12
60 V/cm	72	0	3.45 ± 0.01	26.01 ± 0.05	0.37 ± 0.03	4.10 ± 0.11
		60	3.44 ± 0.00	26.03 ± 0.02	0.36 ± 0.07	4.19 ± 0.08
	48	0	3.52 ± 0.01	25.36 ± 0.23	0.47 ± 0.02	4.26 ± 0.06
		20	3.53 ± 0.00	25.32 ± 0.39	0.46 ± 0.09	4.38 ± 0.15
	36	0	3.54 ± 0.01	24.56 ± 0.21	0.49 ± 0.01	5.28 ± 0.08
		20	3.54 ± 0.01	24.55 ± 0.11	0.52 ± 0.09	5.17 ± 0.02
	24	0	3.56 ± 0.00	24.45 ± 0.42	0.36 ± 0.01	5.39 ± 0.31
		20	3.55 ± 0.00	24.55 ± 0.18	0.37 ± 0.04	5.41 ± 0.19
	18	0	3.58 ± 0.01	24.43 ± 0.43	0.28 ± 0.07	5.35 ± 0.03
		20	3.57 ± 0.01	24.33 ± 0.12	0.27 ± 0.01	5.42 ± 0.10

^a^Results are expressed as means ± SD. Values in the same column are not significantly different (*P* > 0.05)

^b^Color values are L* (lightness), a* (redness), and b* (yellowness)

Although ohmic heating is no longer regarded as a new technology, target microbe reductions have to be assessed in new application environments which include product type and production setting. In this study, optimized voltage gradient and juice concentration for ohmic heating gave a distinct advantage in terms of both bactericidal and economic aspects but also ensured minimal quality loss. However, since ohmic heating was performed in a small-scale batch system, energy and performance criteria have limited significance relative to larger-scale processing units. Therefore, further research incorporating more sophisticated experimental conditions to industrial-scale continuous systems is needed.

## Conclusions

Novel thermal processing interventions employed by the fruit juice industry for controlling foodborne pathogens involve the utilization of sophisticated systems, which enable reduced processing times and temperatures to prevent loss of nutritional and sensory quality while still securing outstanding bactericidal efficacy. Ohmic heating is one of the most promising thermal technologies for effectively inactivating foodborne pathogens in this respect. In the present study, the optimum processing parameters of ohmic heating treatment such as applied voltage gradients and °Brix of apple juice concentrates were investigated to provide benefits with regard to bactericidal, sensory, and economic aspects. These results can be utilized by the apple juice industry for effective application of ohmic heating.

## Abbreviations

ATCC: American type culture collection
CFU: Colony forming unit
OAB: Oxford agar base
PW: Peptone water
SMAC: Sorbitol MacConkey agar
SPC: System performance coefficient
TSA: Tryptic soy agar
TSB: Tryptic soy broth
VAC: Volts alternating current
XLD: Xylose lysine desoxycholate agar

## References

[1] Feng P (1995). *Escherichia coli* O157:H7: novel vehicles of infection and emergence of phenotypic variants. Emerg Infect Dis.

[2] Sung HJ, Song WJ, Kim KP, Ryu S, Kang DH (2014). Combination effect of ozone and heat treatments for the inactivation of *Escherichia coli* O157:H7, *Salmonella* Typhimurium, and *Listeria monocytogenes* in apple juice. Int J Food Microbiol.

[3] Mihajlovic B, Dixon B, Couture H, Farber J (2013). Qualitative microbiological risk assessment of unpasteurized fruit juice and cider. International Food Risk Analysis Journal.

[4] Cody SH, Glynn MK, Farrar JA, Cairns KL, Griffin PM, Kobayashi J, Fyfe M, Hoffman R (1999). An outbreak of *Escherichia coli* O157:H7 infection from unpasteurized commercial apple juice. Ann Intern Med.

[5] Jain S, Bidol SA, Austin JL, Berl E, Elson F, Lemaile-Williams M, Deasy M, Moll ME (2009). Multistate outbreak of *Salmonella* Typhimurium and Saint-paul infections associated with unpasteurized orange juice–United States, 2005. Clin Infect Dis.

[6] U.S. Food and Drug Administration (1998). Hazard analysis and critical control points (HACCP); procedures for the safe and sanitary processing and importing of juice. Federal Register.

[7] Kroll RG, Patchett RA (1992). Induced acid tolerance in *Listeria monocytogenes*. Lett Appl Microbiol.

[8] Kenney SJ, Burnett SL, Beuchat LR (2001). Location of *Escherichia coli* O157:H7 on and in apples as affected by bruising, washing, and rubbing. J Food Prot.

[9] Roering AM, Luchansky JB, Ihnot AM, Ansay SE, Kaspar CW, Ingham SC (1999). Comparative survival of *Salmonella typhimurium* DT 104, *Listeria monocytogenes*, and *Escherichia coli* O157:H7 in preservative-free apple cider and simulated gastric fluid. Int J Food Microbiol.

[10] U.S. Food and Drug Administration (2004). Guidance for Industry: Juice HACCP Hazards and Controls Guidance First Edition; Final Guidance.

[11] Aguilar-Rosas SF, Ballinas-Casarrubias ML, Nevarez-Moorillon GV, Martin-Belloso O, Ortega-Rivas E (2007). Thermal and pulsed electric fields pasteurization of apple juice : Effects on physicochemical properties and flavour compounds. J Food Eng.

[12] Choi LH, Nielsen SS (2004). The effects of thermal and nonthermal processing methods on apple cider quality and consumer acceptability. J Food Qual.

[13] Pereira RN, Vicente AA (2010). Environmental impact of novel thermal and non-thermal technologies in food processing. Food Res Int.

[14] Lee SY, Sagong HG, Ryu S, Kang DH (2012). Effect of continuous ohmic heating to inactivate *Escherichia coli* O157:H7, *Salmonella* Typhimurium, and *Listeria monocytogenes* in orange juice and tomato juice. J Appl Microbiol.

[15] Sastry SK, Barach JT (2000). Ohmic and inductive heating. J Food Sci.

[16] Castro I, Teixeira JA, Salengke S, Sastry SK, Vicente AA (2003). The influence of field strength, sugar and solid content on electrical conductivity of strawberry products. J Food Process Eng.

[17] Icier F, Ilicali C (2005). The effects of concentration on electrical conductivity of orange juice concentrates during ohmic heating. Eur Food Res Technol.

[18] Park IK, Kang DH (2013). Effect of electropermeabilization by ohmic heating for inactivation of *Escherichia coli* O157:H7, *Salmonella enterica* Serovar Typhimurium, and *Listeria monocytogenes* in buffered peptone water and apple juice. Appl Environ Microbiol.

[19] Lee SY, Ryu SR, Kang DH (2013). Effect of frequency and waveform on inactivation of *Escherichia coli* O157:H7 and *Salmonella enterica* serovar Typhimurium in salsa by ohmic heating. Appl Environ Microbiol.

[20] Chen Z, Zhu C, Zhang Y, Niu D, Du J (2010). Effects of aqueous chlorine dioxide treatment on enzymatic browning and shelf-life of fresh-cut asparagus lettuce (*Lactuca sativa* L.). Postharvest Biol Technol.

[21] Huixian S, Shuso K, Jun-ichi H, Kazuhiko I, Tatsuhiko W, Toshinori K (2007). Effects of ohmic heating on microbial counts and denaturation of proteins in milk. Food Sci Technol Res.

[22] Palaniappan S, Sastry SK (1991). Electrical conductivity of selected juices : influences of temperature, solids content, applied voltage, and particle size. J Food Process Eng.

